# Peripheral Inflammation Enhances Microglia Response and Nigral Dopaminergic Cell Death in an *in vivo* MPTP Model of Parkinson’s Disease

**DOI:** 10.3389/fncel.2018.00398

**Published:** 2018-11-06

**Authors:** Irene García-Domínguez, Karolina Veselá, Juan García-Revilla, Alejandro Carrillo-Jiménez, María Angustias Roca-Ceballos, Marti Santiago, Rocío M. de Pablos, José L. Venero

**Affiliations:** Instituto de Biomedicina de Sevilla (IBiS), Hospital Universitario Virgen del Rocío/CSIC/Universidad de Sevilla and Departamento de Bioquímica y Biología Molecular, Facultad de Farmacia, Universidad de Sevilla, Seville, Spain

**Keywords:** Parkinson’s disease, peripheral inflammation, microglia, lipopolysaccharide, neuroinflammation, MPTP, galectin-3, dopaminergic neurons

## Abstract

The impact of systemic inflammation in nigral dopaminergic cell loss remains unclear. Here, we have investigated the role of peripheral inflammation induced by systemic lipopolysaccharide (LPS) administration in the MPTP-based model of Parkinson’s disease. Brain inflammation, microglia and astroglia activation, disruption of the blood–brain barrier (BBB) and integrity of the nigrostriatal dopaminergic system were evaluated in response to i.p. injection of LPS, MPTP or the combination of both. Our results showed that combinative treatment exacerbates microglia activation and enhances (i) the appearance of galectin-3-positive microglia, recently identified as microglial disease-associated phenotypic marker, (ii) the up-regulation of pro-inflammatory cytokines, (iii) the occurrence of A1 neurotoxic astrocytes, (iv) the breakdown of the BBB, and (v) the loss of dopaminergic neurons in the substantia nigra. Microglia activation was triggered earlier than other degenerative events, suggesting that over-activation of microglia (including different polarization states) may induce dopaminergic neuron loss by itself, initiating the endless cycle of inflammation/degeneration. Our study revitalizes the importance of peripheral inflammation as a potential risk factor for Parkinson’s disease and raises the possibility of using new anti-inflammatory therapies to improve the course of neurodegenerative diseases, including those directly aimed at modulating the deleterious activity of disease-associated microglia.

## Introduction

Parkinson’s disease (PD) is the second most prevalent neurodegenerative disorder after Alzheimer’s disease (AD), which is characterized by a permanent and selective loss of dopaminergic neurons in the substantia nigra (SN) pars compacta (Obeso et al., 2000), with the subsequent loss of dopamine (DA) in the striatum. This loss of DA accounts for many of the symptoms that accompany the disease, including motor dysfunction, mood alterations and cognitive impairment (Olanow et al., 2003). Mitochondrial dysfunction, oxidative stress, excitotoxicity, alterations in the ubiquitin-proteasome system and neuroinflammatory mechanisms have been shown to cooperate in the progressive death of the dopaminergic neurons (Dexter and Jenner, 2013). In this regard, clinical and experimental evidence suggests that PD is associated with neuroinflammatory processes such as microglia activation, T-leukocyte infiltration, and blood–brain barrier (BBB) dysfunction (McGeer and McGeer, 2004; Hirsch and Hunot, 2009; Tiwari and Pal, 2017). Microglia-associated inflammation occurs in different animal models of PD, including those using 1-methyl-4-phenyl-1,2,3,6 tetrahydropyridine (MPTP), 6-hydroxydopamine, lipopolysaccharide (LPS) or rotenone (Castano et al., 1998; Liberatore et al., 1999; Betarbet et al., 2000; Cicchetti et al., 2002; Barnum and Tansey, 2010). More importantly, epidemiological studies have demonstrated that regular users of non-steroidal anti-inflammatory drugs or cyclo-oxygenase inhibitors have about 50% lower PD and AD risk than non-users (Chen et al., 2003, 2005; Esposito et al., 2007; Subramaniam and Federoff, 2017).

Despite the existence of several theories that attempt to explain how nigral dopaminergic neurons die in PD, the etiology of the disease and its possible cure, is far from been understood. Consequently, identification of new PD risk factors is mandatory. In the same vein, a deleterious role for peripheral inflammation in different neurodegenerative diseases is becoming evident (Herrera et al., 2015). For instance, aged persons exposed to systemic infections have a twofold increased risk of AD (Tilvis et al., 2004). Furthermore, induction of a systemic inflammatory response led to reactivation in animal models of multiple sclerosis (Serres et al., 2009). Moreover, Cunningham et al. (2005) showed that systemic inflammation sensitizes microglia to switch to an over-activated pro-inflammatory state in a model of prion disease.

The aim of the present work was to demonstrate whether peripheral inflammation increases neuroinflammation and subsequent neuronal death in the MPTP-based model of PD. Systemic inflammation was firstly induced by a single intraperitoneal injection of LPS. We show for the first time that LPS-induced peripheral inflammation and MPTP act synergistically to enhance (a) the appearance of galectin-3 expressing microglia, recently identified as a phenotypic marker of disease-associated microglia (Keren-Shaul et al., 2017; Krasemann et al., 2017; Mathys et al., 2017), (b) central inflammatory response, and (c) the disruption of the BBB. In addition, systemic inflammation increases vulnerability of dopaminergic neurons to MTP-induced neurodegeneration *in vivo*.

## Materials and Methods

### Animals and Treatments

Male C57BL/6 mice (20–25 g) were used for these studies. They were housed in groups of 4–6, at constant room temperature of 22 ± 1°C and relative humidity (60%), with a 12-h light-dark cycle and free access to food and water. Experiments were carried out in accordance with the Guidelines of the European Union Directive (2010/63/EU) and Spanish regulations (BOE 34/11370-421, 2013) for the use of laboratory animals; the study was approved by the Scientific Committee of the University of Seville. Experiments using MPTP were carried out following the operating instructions about the use, handling and storage of chemical agents of the Prevention Service of the University of Seville (SEPRUS^1^).

Acute MPTP models have been associated to strong inflammatory response in the nigro-striatal dopaminergic system (Kavanagh et al., 2015). Main MPTP acute treatment paradigm is based on four consecutive MPTP injections (16 mg/kg every 2 h). However, the combination of LPS and this acute MPTP paradigm abruptly increased early death rate. To solve this issue, we took advantage of a single injection of MPTP at higher dose (40 mg/kg), which has been shown to cause a selective death of nigral dopaminergic neurons (Mejías et al., 2006). Using this MPTP paradigm, Lopez-Barneo and colleagues demonstrated a 65% decrease of striatal dopaminergic terminals, a 48% loss of nigral TH-positive neurons and a 24% loss of Nissl-stained cells in the SN evaluated 1 week after neurotoxin injection (Mejías et al., 2006). These findings demonstrate neurodegenerative events of the nigro-striatal dopaminergic system and make this MPTP paradigm suitable to test whether or not the presence of peripheral inflammation affects nigral dopaminergic vulnerability.

Crews and colleagues demonstrated that a single dose of 5 mg/kg (i.p.) causes neuroinflammation and delayed death of nigral dopaminergic neurons (starting 7 months after LPS injection) (Qin et al., 2007). Besides, it is well-documented that very little peripheral LPS enters to the brain due to the poor passage through the BBB (Nadeau and Rivest, 1999), Consequently, to full validate the solely contribution of peripheral inflammation in MPTP-induced neurotoxicity, we used a LPS dose 2.5-fold lower than that used by Qin et al. (2007). Consequently, animals were distributed within groups according to treatments, time points and method of analysis: The V (vehicle, control) group receiving a single i.p. injection of both vehicles; the MPTP group, receiving a single i.p. dose of MPTP (40 mg/kg in 0.9% sterile saline; Sigma-Aldrich, St. Louis, MO, United States, Catalogue # M0896); the LPS group receiving a single i.p. LPS injection (2 mg/kg in 0.9% sterile saline; Sigma-Aldrich, St. Louis, MO, United States, Catalogue # L4391), and the LPS/MPTP group receiving a single i.p. dose of MPTP and LPS. In the case of using both treatments, LPS was administered 120 min before the MPTP administration. All treatments were administrated in a volume of 100 μl per 25 g of body weight. At least three animals per group were used (Table 1).

**Table 1:** Number of cases used for each group and each technique.

	Number of animals							
	Striatum				Substantia Nigra			
Technique	Control	LPS	MPTP	LPS + MPTP	Control	LPS	MPTP	LPS + MPTP
IHC: Iba1	3	4	4/3	3	4	4/3	6/3/3	5/4/3
IF: Iba1^+^/Gal3^+^	4	4	4	3	3	3	4	3
qPCR	5	5	5/4/3	5/4/3	5	5/4	5/4/3	6/4/3
IHC: IgG	3	3	4	3	3	3	3	3
TH counting	–	–	–	–	3	3	5	5
HPLC	4	4	3	3	–	–	–	–

Animals were sacrificed at different time points depending on the technique assayed. For immunohistochemistry analysis, animals were sacrificed at 12 h, 24 h, and 2 weeks. For qPCR assays, animals were sacrificed at 12 h. For HPLC and stereological analysis, animals were sacrificed at 2 weeks.

### Immunohistological Evaluation: Tyrosine Hydroxylase (TH), Glial Fibrillary Acidic Protein (GFAP) and Iba-1

At required day and hour after the treatment, animals dedicated to the immunohistochemistry were anesthetized with ketamine (50 mg/kg, Ketamidor^®^, Richter Pharma, Wels, Austria) and medetomidine (10 mg/kg, Domtor^®^, Ecuphar, Oostkamp, Belgium) and perfused through the heart with 0.9% saline followed by 40 ml of 4% paraformaldehyde in phosphate buffer, pH 7.4. Brains were removed and then cryoprotected serially in sucrose in PBS, pH 7.4, first in 10% (24 h), then in 20% sucrose (24 h), and then in 30% sucrose until sunk (2–5 days). The brains were then frozen in isopentane at -40°C (10 min) and kept at -40°C. We analyzed early (12 and 24 h) and delayed response (2 weeks) after challenge.

Thaw-mounted 25-μm coronal sections were cut on a cryostat at -15°C and mounted in gelatin-coated slides. Primary antibodies used were rabbit-derived anti-tyrosine hydroxylase (anti-TH, Sigma, 1:300), mouse-derived anti-glial fibrillary acidic protein (anti-GFAP; Sigma, 1:400) and rabbit-derived anti-Iba-1 (Wako, 1:500). Incubations and washes for all the antibodies were in Tris-buffered saline (TBS), pH 7.4. All work was done at room temperature. Sections were washed and then treated with 0.3% hydrogen peroxide in methanol for 20 min, washed again, and incubated in a solution containing TBS and 1% goat serum (Vector) for 60 min in a humid chamber. Slides were drained and further incubated with the primary antibody (Table 2) in TBS containing 1% goat serum and 0.25% Triton-X-100 for 24 h. Sections were then incubated for 2 h with biotinylated goat anti-rabbit IgG (Vector, 1:200). The secondary antibody was diluted in TBS containing 0.25% Triton-X-100, and its addition was preceded by three 10-min rinses in TBS. Sections were then incubated with ExtrAvidin^®^-Peroxidase solution (Sigma, 1:100). The peroxidase was visualized with a standard diaminobenzidine/hydrogen peroxide reaction for 6 min (do not ingest and breathe dust).

**Table 2:** Antibodies for immunofluorescence, immunohistochemistry and Western Blot.

Antibody	Catalog number	
Rabbit-derived anti-TH	T8700	SIGMA
Mouse-derived anti-GFAP	G3893-100UL	SIGMA
Rabbit-derived anti-Iba1	019-19741	Wako
Goat-derived anti-mouse biotinylated	BA 9200	Vector
Goat-derived anti-occludin	SC-8145	Santa Cruz Biotechnology Inc.
Rat-derived anti-C3	SC-58926	Santa Cruz Biotechnology Inc.
Sheep-derived anti-TH	NB300-110	NOVUS
Rabbit-derived anti-Caspase 3	9662S	Cell signaling
Goat-derived anti-Galectin 3	AF-1197	R&D Systems
Alexa Fluor 647 anti-mouse	A31571	Invitrogen
Alexa Fluor 488 anti-rat	A21208	Invitrogen
Alexa Fluor 488 anti-goat	A11055	Invitrogen
Alexa Fluor 546 anti-rabbit	A10040	Invitrogen
Alexa Fluor 488 anti-rabbit	A21206	Invitrogen
Alexa Fluor 647 anti-sheep	A-21448	Invitrogen
Goat-derived anti-rabbit biotinylated	BA 1000	Vector
Goat-derived anti-mouse biotinylated	BA 9500	Vector

### Immunohistological Evaluation of BBB Disruption

Breakdown of the BBB was assessed by employing a one-step immunohistochemical detection of IgG with some modifications (Tomas-Camardiel et al., 2004). Briefly, sections were incubated for 2 h in biotinylated horse anti-mouse IgG (Vector; 1:200; Table 2) in TBS containing 1% bovine serum albumin (BSA) and 0.2% (Triton-X-100). Visualization of IgG immunoreactivity was identical to that described above for immunohistochemistry.

### Immunofluorescence

Animals were perfused and sections were prepared as described above. Incubations and washes for all the antibodies were in PBS, pH 7.4. All work was done at room temperature, unless otherwise noted. Sections were blocked with PBS containing 5% BSA for 2 h. The slides were then incubated overnight at 4°C with the primary antibody (Table 2): goat-derived anti-occludin (Santa Cruz Biotechnology Inc.; 1:50), mouse-derived anti-GFAP (Sigma; 1:400), rat-derived anti-C3 (Santa Cruz Biotechnology Inc.; 1:50), sheep-derived anti-TH [NOVUS (Biomol); 1:1000], rabbit-derived anti-caspase 3 (Cell Signaling; 1:250), rabbit-derived anti-Iba1 (Wako; 1:1000) and goat-derived anti-galectin-3 (R&D Systems; 1:250). Primary antibodies were diluted in PBS containing 1% BSA and 1% Triton X-100. After three washes in PBS, sections were incubated with (Table 2) secondary antibodies conjugated to fluorescein (Vector; 1:300), Alexa Fluor^®^ 488 and Alexa Fluor^®^ 647 (Invitrogen; 1:200) for 2 h at room temperature in the dark. Fluorescence images were acquired using a confocal laser scanning microscope (Zeiss LSM 7 DUO) and processed using the associated software package (ZEN, 2010).

### Immunohistochemistry Data Analysis

For GFAP immunoreactivity, the AnalySIS imaging software (Soft Imaging System GmbH, Münster, Germany) coupled to a Polaroid DMC camera (Polaroid, Cambridge, MA, United States) attached to a Leika light microscope (Leica Mikroskopie, Wetzlar, Germany) was used. Quantification of Iba-1 and TH positive cells in the SN was estimated according to a modified stereological approach. In each animal, every seventh section was used with random starting points and systematically distributed through the anteroposterior axis of the analyzed region. For counting cells showing Iba-1 immunoreactivity, a systematic sampling of the area occupied by the Iba-1 positive cells in each section was made from a random starting point with a grid adjusted to count five fields per section. An unbiased counting frame of known area (40 μm × 25 μm = 1000 μm^2^) was superimposed on the tissue section image under a 100× oil immersion objective. The different types of Iba-1-positive cells (displaying different shapes depending on their activation state) were counted as a whole and expressed as cells per mm^2^. The number of TH-positive neurons in the SN was estimated using a fractionator sampling design (Gundersen et al., 1988). Counts were made at regular predetermined intervals (*x* = 150 μm and *y* = 200 μm) within each section. An unbiased counting frame of known area (15686,7 μm^2^) was superimposed on the tissue section image under a 40× objective. Therefore, the area sampling fraction was 15686,7 μm^2^/63380,6 μm^2^ = 0.248. In all animals, 25-μm sections, each 175 μm apart, were analyzed; thus, the fraction of sections sampled was 25/175 = 0.143. The total number of neurons in the SN was estimated by multiplying the number of neurons counted within the sample regions by the reciprocals of the area sampling fraction and the fraction of section sampled.

Relative amount of IgG extravasation was measured using Image-J software (downloaded as a free software package from the public domain^2^). Areas of IgG occupancy were counted using three randomly distributed fields per section and three sections per animal. The system allows defining a threshold to discern between IgG immunoreactivity and background and the percentage of IgG occupancy measured.

Iba-1/galectin-3 co-localizing cells were counted with Image-J software using the plugin *cell counter.* At least three animals were used for each condition and number of Iba-1/galectin-3 cells were measure in a minimum of three eyespot images acquired using a confocal laser scanning microscope (Zeiss LSM 7 DUO).

### Measurement of DA by High-Performance Liquid Chromatography (HPLC)

Analysis of striatal dopamine was performed by HPLC with electrochemical detection. A Merck L-6200A intelligent pump was used in conjunction with a glassy carbon electrode set at -550 mV (DECADE II, ANTEC, Netherlands). A Merck Lichrocart cartridge (125 mm × 4 mm) column filled with Lichrospher reverse-phase C_18_ 5 μm material was used. The mobile phase consisted of a mixture of 0.05 M of sodium acetate, 0.4 mM of 1-octanesulfonic acid, 0.3 mM of Na_2_EDTA and 70 ml methanol/l, adjusted to pH 4.1 with acetic acid. All reactive agents and water were of HPLC grade. The flow rate was 1.0 ml/min. Measurement of DA in fresh tissue was performed according to the method previously described (Ismaiel et al., 2016). Concentrations of striatal DA samples were calculated with the aid of the eDAQ PowerChrom 280 software.

### Real Time RT-PCR

The results on microglial activation in terms of Iba-1 immunoreactivity showed that the highest activation takes places after 12 h of treatment. Therefore, animals designated to RT-PCR were perfused with saline sacrificed by decapitation 12 h after the treatment. Brains were then removed and striatum and SN were dissected, snap frozen in liquid nitrogen and stored at -80°C. Real Time RT-PCR was performed essentially as described previously (Sanchez-Hidalgo et al., 2016). Total RNA was extracted from the SN using RNeasy^®^ Mini kit (Qiagen). cDNA was synthesized from 1 μg of total RNA using RevertAid First Strand cDNA Synthesis Kit (Thermo Fisher Scientific) in 10 μl reaction volume as described by the manufacturer. Real-time PCR was performed with SensiFAST^TM^ SYBR No-ROX Kit (Bioline), 0.4 μM primers and 4.2 μl cDNA. Controls were carried out without cDNA. Amplification was run in a LightCycler 480 (Roche Molecular Systems) thermal cycler at 95°C for 2 min followed by 40 cycles of 95°C for 5 s and 60°C for 15 s. Following amplification, a melting curve analysis was performed by heating the reactions from 65 to 95°C in 0.1°C/s while monitoring fluorescence. Analysis confirmed a single PCR product at the melting temperature. β-actin served as reference gene and was used for samples normalization. Primer sequences for β-actin, tumor necrosis factor (TNF)-α, interferon (INF)-β, interleukin (IL)-1β and IL-6 are shown in Table 3. The cycle at which each sample crossed a fluorescence threshold (*C*t) was determined, and the triplicate values for each cDNA were averaged. Analyses of real-time PCR were done using a 2*^C^*^t^ relative quantification method.

**Table 3:** Primers used for real-time PCR.

ARNm target	Primers sense (S) and antisense (A) sequences	Temp (°C)
**β-Actin**	S:5′- GGCTATGCTCTCCCTCACG A:5′- CTTCTCTTTGATGTCACGCACG	60
**TNF-α**	S:5′- TGCCTATGTCTCAGCCTCTTC A: 5′- GAGGCCATTTGGGAACTTCT	60
**INF-β**	S:5′- AACTTCCAAAACTGAAGACC A:5′- AACTCTGTTTTCCTTTGACC	60
**IL-6**	S:5′- GACAAAGCCAGAGTCCTTCAGA A:5′- AGGAGAGCAATTGGAAATTGGGG	60
**IL-1β**	S:5′- TGTAATGAAAGACGGCACACC A:5′- TCTTCTTTGGGTATTGCTTGG	60

### Statistical Analysis

Results are expressed as mean ± SD. Means were compared by one-way ANOVA followed by the Fisher’s LSD test for *post hoc* multiple range comparisons. An alpha level of 0.05 was used. The Statgraphics Plus 3.0 statistical package was used for the analyses.

## Results

### Quantification of Microglia

**FIGURE 1:**
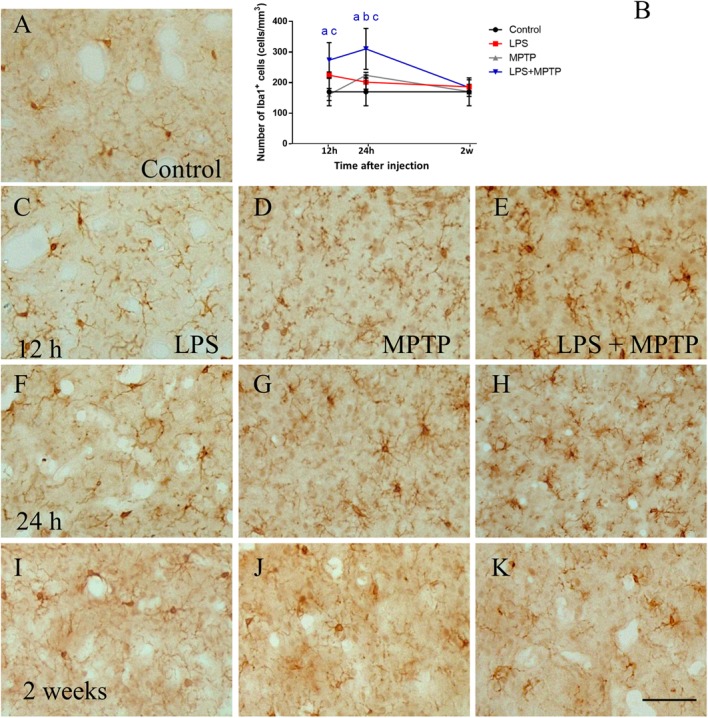
Effect of LPS and MPTP on the activation of microglia in the striatum. Coronal sections showing Iba-1 immunoreactivity in the striatum after the different treatments assayed. **(A)** Control animals. **(C,F,I)** Animals treated with LPS during 12 h, 24 h, and 2 weeks, respectively. **(D,G,J)** Animals treated with MPTP during 12 h, 24 h, and 2 weeks, respectively. **(E,H,K)** Animals treated with both LPS and MPTP during 12 h, 24 h, and 2 weeks, respectively. Note that LPS highly increases the microglia activation response to MPTP injection. Scale bar: 100 μm. **(B)** Quantification of Iba-1-positive cells in the striatum of rats. Results are mean ± SD of at least three independent experiments and are expressed as cell/mm^2^. One-way ANOVA followed by the Fischer’s LSD *post hoc* test for multiple comparisons was used for statistical analysis, with α = 0.05: a, compared with the control; b, compared with the LPS group; c, compared with the MPTP group; *p* < 0.001.

Iba-1 is a protein associated with major histocompatibility complex II, which is expressed in all microglial phenotypes but overexpressed in reactive microglia. Hence, we first used Iba-1 immunohistochemistry to evaluate the number and morphology of microglial cells. In striatum, control animals showed a faint Iba1 immunostaining characterized by a small number of highly-ramified microglial cells (170.2 cell/mm^2^ ± 45.6; Figures 1A,B). Treatment with LPS alone induced a transient activation of microglia that peaked after 12 h (224.5 ± 10.3 cells/mm^2^; *p* < 0.001; Figures 1B,C,F,I). In this brain structure, microglial cells were bigger and exhibited thicken processes in response to LPS treatment. The treatment with MPTP alone showed a time-course characterized by a significant increase after 24 h (161.5 ± 19.6 and 224.2 ± 9.7 cell/mm^2^, respectively, *p* < 0.001; Figures 1B,D,G,J). This MPTP-induced effect over microglial cells was significantly enhanced when LPS was administered 12 h prior to MPTP (273.8 ± 56,9 cell/mm^2^; *p* < 0.001; Figures 1B,E,H,K). The results demonstrate how peripheral inflammation modulates microglia brain response during neurodegeneration. Two weeks after treatment, number and morphology of most of microglial cells were similar to those found in controls. Still, some microglial cells in the striatum from LPS/MPTP- and MPTP-treated animals showed a round, phagocytic morphology without processes, features of chronically activated microglia (Figure 1K).

**FIGURE 2:**
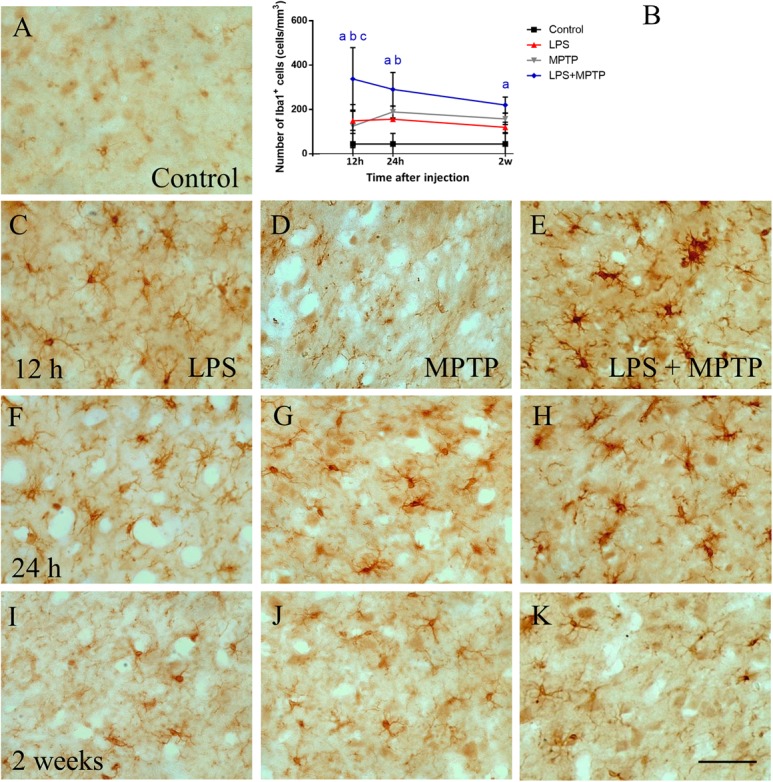
Effect of LPS and MPTP on the activation of microglia in the SN. Coronal sections showing Iba-1 immunoreactivity in the SN. **(A)** Control animals. **(C,F,I)** Animals treated with LPS during 12 h, 24 h, and 2 weeks, respectively. Note that LPS increase microglial activation, especially after 12 h. **(D,G,J)** Animals treated with MPTP during 12 h, 24 h, and 2 weeks, respectively. **(E,H,K)** Animals treated with both LPS and MPTP during 12 h, 24 h, and 2 weeks, respectively. Note that LPS highly increases the microglia activation response to MPTP injection. Scale bar: 50 μm. **(B)** Quantification of Iba-1-positive cells in the SN of rats. Results are mean ± SD of at least three independent experiments and are expressed as cell/mm^2^. One-way ANOVA followed by the Fischer’s LSD *post hoc* test for multiple comparisons was used for statistical analysis, with α = 0.05: a, compared with the control; b, compared with the LPS group; c, compared with the MPTP group; *p* < 0.001.

Similar results were found when microglia were analyzed in the SN. Strikingly, LPS treatment alone showed increased density of microglial cells that peaked again 12 h after exposition (156.2 ± 9.4; *p* < 0.0001; Figures 2B,C,F,I) compared to controls (44.9 ± 47.2 cell/mm^2^, Figures 2A,B). In MPTP-treated animals, an increase in the number of microglial cells was found at 24 h (173.5 ± 0.8 cells/mm^2^; *p* < 0.0001; Figures 2B,D,G,J). Remarkably, LPS/MPTP-injected animals showed a stronger microglia activation compared with animals treated with LPS or MPTP alone, this effect being especially evident at 12 h (337.9 ± 140.8 cells/mm^2^; *p* < 0.0001; Figures 2B,E,H,K). These data demonstrate again a synergistic effect between peripheral and central inflammation.

In the striatum, conversely, a significant number of microglial cells was seen in the SN of MPTP-injected animals (157 ± 25.7 cell/mm^2^), which further increased in the LPS + MPTP group (220.4 ± 36.0 cells/mm^2^; *p* < 0.0001; Figure 2B,I–K). Microglia exhibited typical morphological features of activated cells with large bodies and thick processes (Figure 2K). These features were even more evident than those seen in striatal tissue (Figure 1K).

**FIGURE 3:**
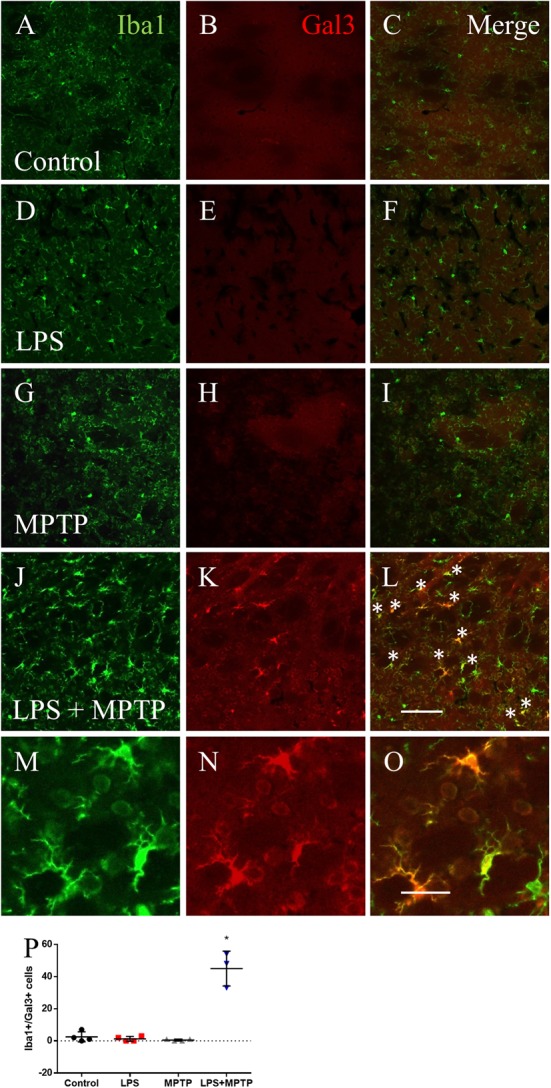
Lipopolysaccharide (LPS)/MPTP induce the appearance of galectin-3 (Gal3)-expressing microglial cells in striatum. Immunofluorescence for Iba1 **(A,D,G,J,M)** and Gal3 **(B,E,H,K,N)** in the different treatment assayed. Only LPS/MPTP group shows a robust expression of Gal3 microglial cells (merged images, **C,F,I,L,O**). Scale bar: 100 μm. **(M–O)** Shows high magnification pictures of Iba-1, Gal3 and merge image of the LPS/MPTP group. Scale bar: 500 μm. **(P)** Quantification of Iba-1/Gal3 co-localizing cells in the different treatment assayed. Results are mean ± SD of at least three independent experiments and are expressed as number of cells. One-way ANOVA followed by the Fischer’s LSD *post hoc* test for multiple comparisons was used for statistical analysis, with α = 0.05: ^∗^, compared with the control; *p* < 0.0001.

**FIGURE 4:**
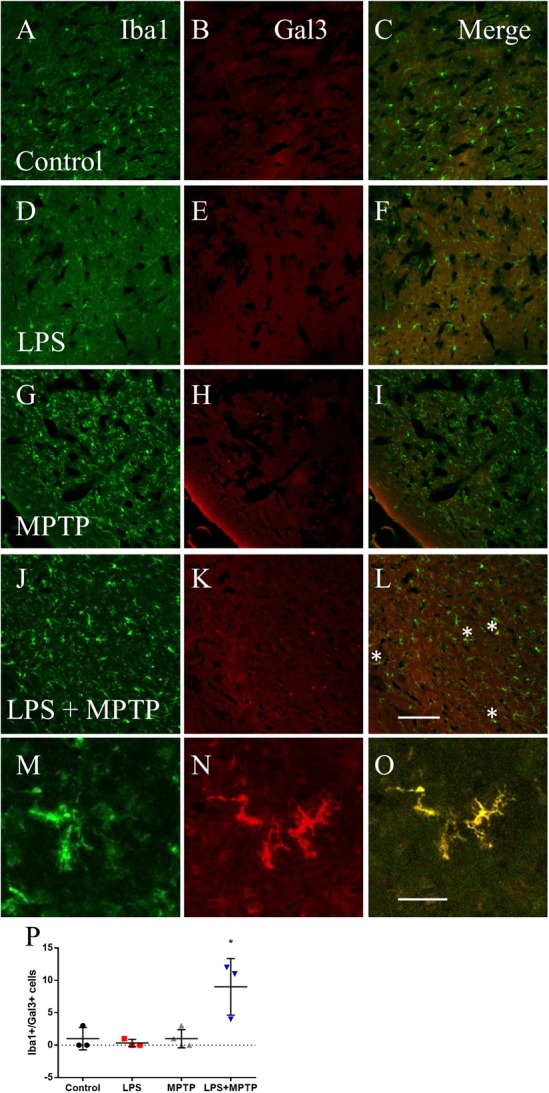
Lipopolysaccharide/MPTP induce the appearance of galectin-3 (Gal3)-expressing microglial cells in SN. Immunofluorescence for Iba1 **(A,D,G,J,M)** and Gal3 **(B,E,H,K,N)** in the different treatment assayed. Only LPS/MPTP group shows a clear expression of Gal3 microglial cells (merged images, **C,F,I,L,O**). Scale bar: 100 μm. **(M–O)** Shows high magnification pictures of Iba-1, Gal3 and merge image of the LPS/MPTP group. Scale bar: 500 μm. **(P)** Quantification of Iba-1/Gal3 co-localizing cells in the different treatment assayed. Results are mean ± SD of at least three independent experiments and are expressed as number of cells. One-way ANOVA followed by the Fischer’s LSD *post hoc* test for multiple comparisons was used for statistical analysis, with α = 0.05: ^∗^, compared with the control; *p* < 0.05.

Recent transcriptomic studies have characterized the molecular signature of microglia under different disease conditions and identified a common disease-associated phenotype (DAM) or microglia neurodegenerative phenotype (Keren-Shaul et al., 2017; Krasemann et al., 2017; Mathys et al., 2017). A selected group of genes were found to be strongly up-regulated in the microglial phenotype associated to neurodegeneration, including Itgax, Clec7a and Lgals3 (galectin-3), which differs from the classically M1-like pro-inflammatory phenotype (Keren-Shaul et al., 2017; Krasemann et al., 2017; Mathys et al., 2017). We have long characterized novel roles associated to galectin-3 within microglial cells under different disease conditions (Boza-Serrano et al., 2014; Burguillos et al., 2015; Yip et al., 2017). Consequently, we analyzed microglial galectin-3 expression under the different experimental conditions. In agreement with previous findings, homeostatic microglia lack galectin-3 expression (Burguillos et al., 2015). Since most significant changes in terms of microglia density and activation, showed by Iba1-labeled microglia, was seen at 12 h after LPS/MPTP, we analyzed the presence of microglial galectin-3 at this time under the different experimental conditions, in both striatum and SN. Systemic LPS or MPTP alone failed to induce microglial galectin-3 expression in both structures. In contrast, in the combinative group, a strong up-regulation of galectin-3 was found in striatum (20-fold vs. control levels; *p* < 0.0001; Figure 3) and SN (6-fold vs. control levels; *p* < 0.01; Figure 4).

Since most relevant microglial changes were found 12 h after LPS/MPTP, we wanted to know if the appearance of microglia polarization toward a neurodegenerative phenotype precedes degenerative events in nigral dopaminergic neurons. Hence, we performed dual confocal immunofluorescence of TH/cleaved caspase-3 (apoptotic marker) in the ventral mesencephalon at 12 and 24 h after the different experimental conditions. No apparent early loss of integrity of nigral dopaminergic neurons was evident in any of the experimental conditions tested, including LPS/MPTP at 12 h (not shown) and 24 h (Supplementary Figure S1). Only under combined LPS/MPTP treatment was evident the appearance of cleaved caspase-3 in the ventral mesencephalon 24 h after. We, however, failed to detect any TH-labeled neuron showing cleaved caspase-3 (Supplementary Figure S1).

Overall, we conclude that systemic inflammation enhances the switch from homeostatic to disease-associated microglia in the nigrostriatal system in response to MPTP treatment preceding the onset of nigrostriatal dopaminergic degeneration.

### Quantification of the Levels of TNF-α, IFN-β, IL-1β, and IL-6 mRNAs

**FIGURE 5:**
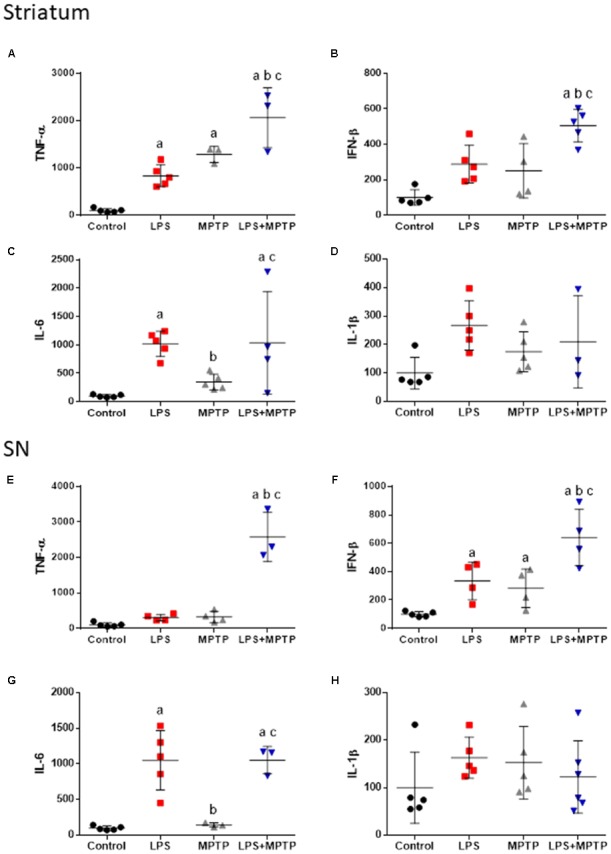
Effect of LPS and MPTP on the expression of TNF-α, IFN-β, IL-1β, and IL-6 mRNAs in the striatum and SN. Quantification of mRNAs expression was made by real-time RT-PCR. Results are mean ± SD of at least three independent experiments, and are expressed as percentage of controls. Statistical signification: One-way ANOVA followed by the LSD *post hoc* test for multiple range comparisons; a, compared with control; b, compared LPS; c, compared with MPTP. **(A)** TNF-α mRNA in striatum, *p* < 0.001; **(B)** INF-β mRNA in striatum, *p* < 0.001; **(C)** IL-6 mRNA in striatum, *p* < 0.01; **(D)** IL-1β mRNA in striatum; **(E)** TNF-α mRNA in SN, *p* < 0.001; **(F)** INF-β mRNA in SN, *p* < 0.001; **(G)** IL-6 mRNA in SN, *p* < 0.001; **(H)** IL-1β mRNA in SN.

We quantified the expression of mRNAs encoding for classical neurotoxic pro-inflammatory markers including TNF-α, IFN-β, IL-1β, and IL-6 in striatum and SN of mice sacrificed 12 h after the different experimental conditions including: control animals, LPS-, MPTP-, and LPS/MPTP-injected animals. Systemic LPS induced a clear pro-inflammatory response in the nigrostriatal system with significant increase in mRNA levels for TNF-α and IL-6 in striatum and IFN-β and IL-6 in the ventral mesencephalon (Figure 5). In contrast, the effect of MPTP alone was negligible. Yet, significant increase of TNF-α expression was found in striatum (Figure 5A) and also IFN- β in SN (Figure 5F). Highest pro-inflammatory response was clearly achieved when LPS and MPTP were combined, with significant increase of TNF-α, IFN-β, and IL-6 in striatum and SN (Figure 5). Statistical analysis demonstrated that combined LPS/MPTP treatment acted synergistically to increase mRNA levels for TNF-α and IFN-β in both striatum and SN (Figure 5).

### Blood–Brain Barrier Impairment

**FIGURE 6:**
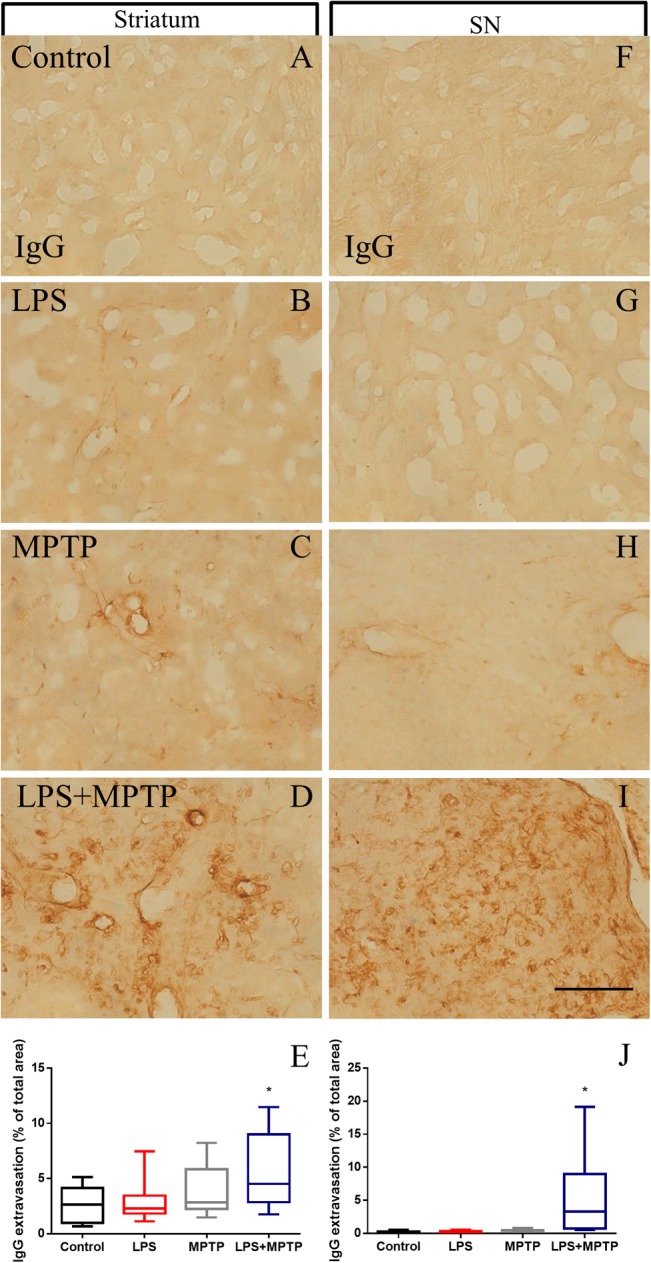
Effect of LPS and MPTP on the BBB integrity in the striatum and SN. Normal pattern of IgG immunoreactivity in control animals in striatum **(A)** and SN **(F)**. Note the absence of IgG immunoreactivity. LPS and MPTP alone failed to induce a significant IgG extravasation in striatum **(B,C)** and SN **(G,H)**. IgG extravasation is, however, strongly induced 12 h after the combination of LPS and MPTP. Note the atypical presence of IgG immunostaining in striatum **(D)** and SN **(I)**. Scale bar: 100 μm. Quantification of the area expressing IgG immunoreactivity in striatum **(E)** and SN **(J)**. Results are mean ± SD of at least three independent experiments and are expressed as % of total area. One-way ANOVA followed by the Fischer’s LSD *post hoc* test for multiple comparisons was used for statistical analysis. ^∗^, different of the rest of the treatment assayed; *p* < 0.05.

We first analyzed the expression pattern of occludin (an integral membrane protein that directly regulates the tight junction paracellular permeability) 12 h after challenge (maximal response of microglia density and activation) (Kuan et al., 2016). We analyzed both, striatum and SN and found a decrease in the expression of this protein in animals treated concomitantly with LPS and MPTP (Supplementary Figure S2). This finding suggests an alteration in the BBB permeability in the nigrostriatal system. Consequently, we next quantitatively analyzed the integrity of the BBB. To achieve this, we studied IgG extravasation into the nigrostriatal system 12 h after challenge. Treatment with LPS or MPTP alone failed to alter BBB integrity in terms of IgG extravasation (Figure 6A–C,E). However, combined LPS/MPTP treatment highly altered BBB integrity in the striatum as demonstrated by IgG extravasation (Figures 6D,E). Similar results were found in SN, where only LPS/MPTP group showed increased immunoreactivity of IgG into the analyzed brain parenchyma (Figures 6F–J). We additionally extended our analysis to other brain areas not directly related to the nigrostriatal system. Thus, we analyzed BBB integrity in cerebral cortex and midbrain (adjacent to the superior colliculus) in response to LPS, MPTP, and LPS + MPTP 12 h after challenge. Interestingly, BBB disruption was again observed in response to LPS + MPTP but not to LPS or MPTP alone in both analyzed areas (Supplementary Figure S3), further supporting that peripheral systemic inflammation and MPTP synergistically enhance/induce BBB breakdown.

### Induction of A1 Astrocytic Phenotype

**FIGURE 7:**
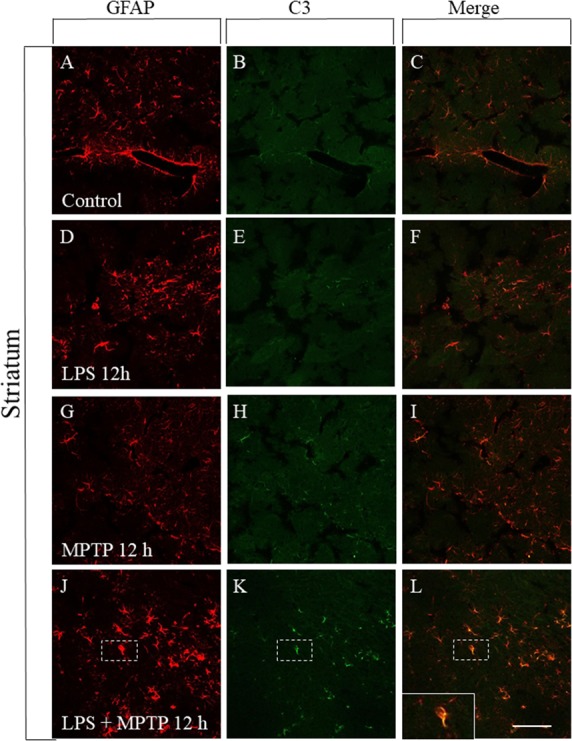
Effect of LPS and MPTP on the A1 astrocytic phenotype in the striatum. Immunofluorescent double labeling of GFAP and C3 shows the absent of C3 immunostaining in non-reactive astrocytes present in the control striatum **(A–C)**. A similar pattern was seen in the striatum of animals treated with LPS **(D–F)** or MPTP **(G–I)** alone. However, the combination of LPS and MPTP clearly induced the expression of A1 astrocytic phenotype in striatum **(J–L)**. Scale bar: 100 μm.

**FIGURE 8:**
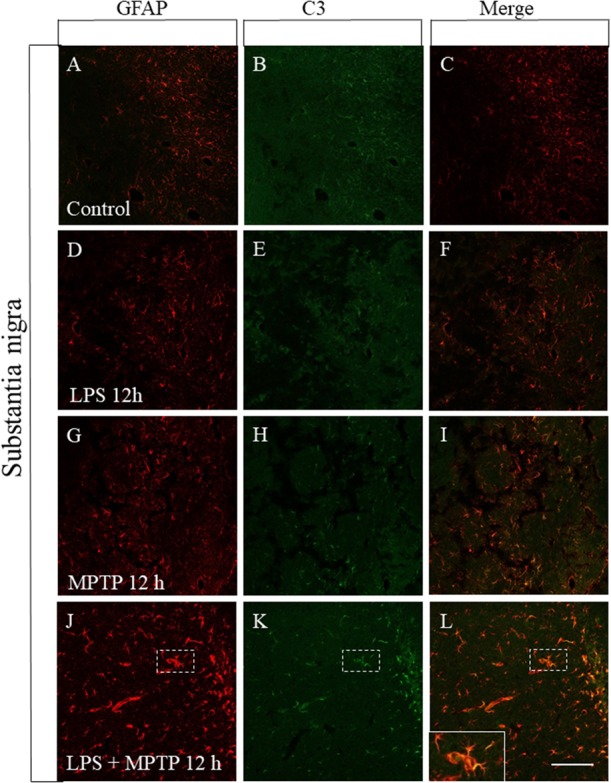
Effect of LPS and MPTP on the A1 astrocytic phenotype in the SN. Immunofluorescent double labeling of GFAP and C3 shows the absent of C3 immunostaining in non-reactive astrocytes present in the control SN **(A–C)**. A similar pattern was seen in the SN of animals treated with LPS **(D–F)** alone. Some neurotoxic astrocytes showing the A1 phenotype are present in the SN of animals treated with MPTP alone **(G–I)**. Again, the combination of LPS and MPTP clearly induced the expression of A1 astrocytic phenotype in SN **(J–L)**. Scale bar: 100 μm.

Reactive astrocytes showing the A1 phenotype have been recently identified and found to be neurotoxic as opposed to A2 astrocytes, which are neuroprotective (Liddelow et al., 2017). Reactive microglia induces reactive A1 astrocyte polarization, which highly up-regulates complement component 3 (C3) expression, in sharp contrast to A2 astrocytes (Liddelow et al., 2017). Therefore, we have performed a double immunofluorescence confocal analysis in order to study the astrocytic phenotype in our experimental conditions. In control animals, astrocytes were not activated and failed to express C3, both in striatum and SN (Figures 7, 8A–C). Treatment with LPS or MPTP alone also failed to induce the A1 phenotype in striatum, at least at the post-injection time analyzed (12 h) (Figures 7D–I). However, the combination of both treatments clearly induced the A1 neurotoxic phenotype in the striatum (Figures 7J–L). In the SN, LPS alone again failed to induce the A1 astrocytic phenotype at 12 h post-injection (Figures 8D–F). However, A1 reactive astrocytes population were minority after the treatment with MPTP alone (Figures 8G–I). In contrast, this phenotype shift was much more evident when LPS and MPTP were combined (Figures 8J–L). Collectively, these data demonstrate that peripheral inflammation early triggers A1 astrocytic phenotype under conditions of dopaminergic neurodegeneration.

### Dopaminergic Neuronal Loss

**FIGURE 9:**
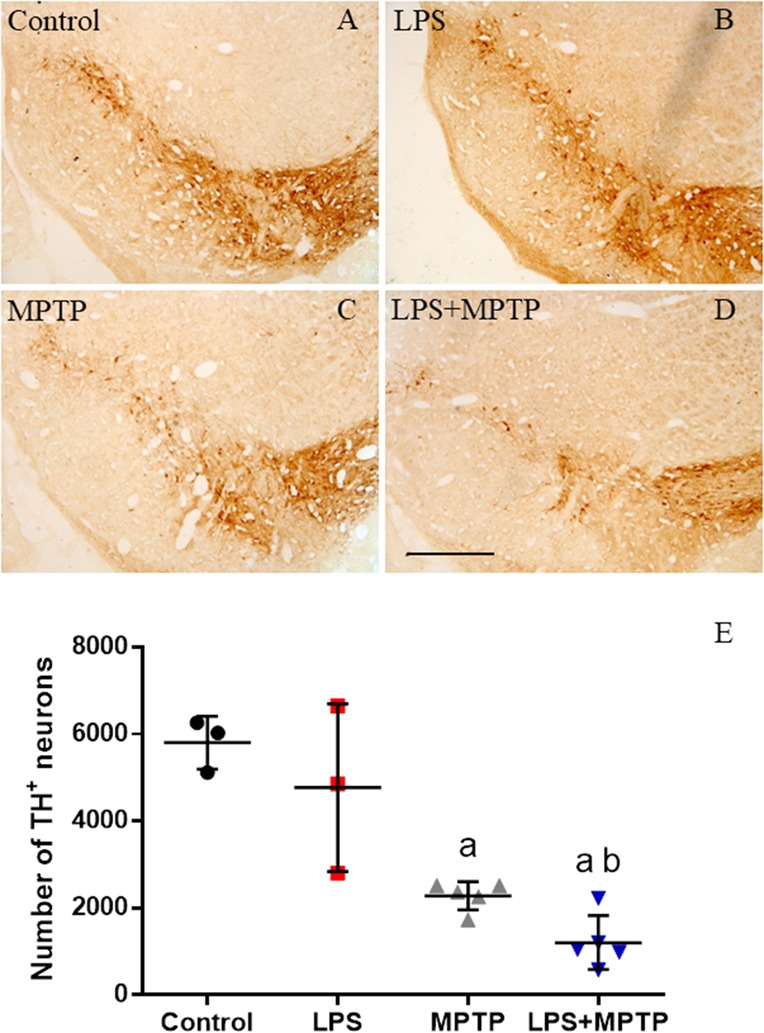
Effect of LPS and MPTP on the dopaminergic neurons. **(A)** Coronal section showing TH immunoreactivity in a control animal. **(B)** TH immunoreactivity 2 weeks after the injection of LPS. No significant changes are observed. **(C)** TH immunoreactivity 2 weeks after the injection of MPTP. There is a clear loss of dopaminergic neurons in the SN. **(D)** TH immunoreactivity 2 weeks after the injection of both LPS and MPTP. The loss of neurons is higher. Scale bar: 500 μm. **(E)** Quantification of the number of TH-positive cells. Results are mean ± SD of three independent experiments, and are expressed as TH-positive cells within the bounded area of the SN. Statistical signification (ANOVA followed by the LSD *post hoc* test for multiple comparisons): a, compared with control; b, compared with MPTP; *p* < 0.001.

We used TH antibody to label dopaminergic neurons in SN of mice treated with LPS, MPTP, combined LPS/MPTP and controls. Analysis performed 2 weeks after the neurotoxin administration showed clear neurodegenerative features. This result was further evaluated by stereology. An even distribution of TH-positive neurons was seen in the SN of control animals (5803 ± 604; Figures 9A,E), showing that the primary antibody was highly selective to dopaminergic neurons. Our stereological analysis of dopaminergic neurons in the SN agrees with previously published reports (Smeyne et al., 2016; Alam et al., 2017; Ip et al., 2017). As expected, MPTP-treated mice showed a significant decrease of 60.7% in the number of dopaminergic neurons of SN (Figures 9C,E, *p* < 0.001). LPS-injected animals failed to induce loss of nigral dopaminergic neurons 2 weeks after the treatment (4770 ± 1924; Figures 9B,E). Strikingly, LPS and MPTP acted synergistically and dopaminergic cell death was significantly increased compared with MPTP alone, reaching 83.6% of cell loss (Figures 9D,E, *p* < 0.001).

**FIGURE 10:**
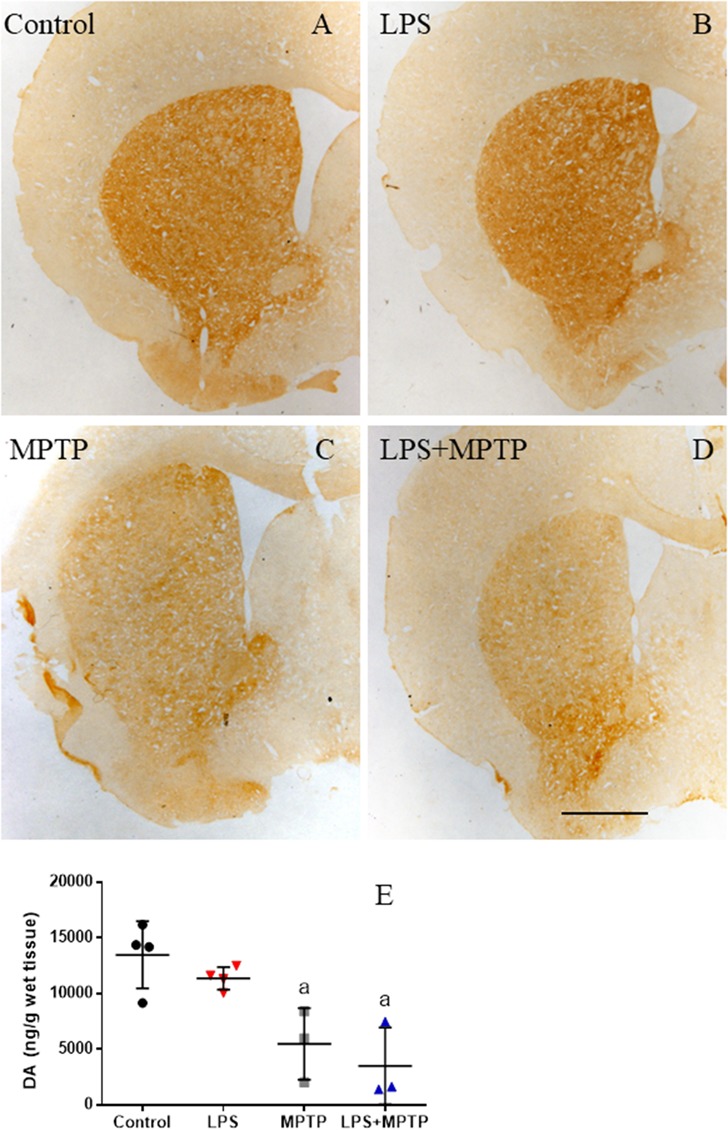
Effect of LPS and MPTP on the dopaminergic terminals. **(A)** Coronal section showing TH immunoreactivity in a control animal. **(B)** TH immunoreactivity 2 weeks after the injection of LPS. No significant changes are observed. **(C)** TH immunoreactivity 2 weeks after the injection of MPTP. There is a clear loss of TH immunoreactivity in the striatum. **(D)** TH immunoreactivity 2 weeks after the injection of both LPS and MPTP. Loss of TH immunoreactivity is higher. Scale bar: 500 μm. **(E)** Amounts of dopamine in the striatum of control and treated animals. Numbers are mean ± SD of at least three independent experiments and are expressed as nanograms per gram of wet tissue.

We also analyzed dopaminergic terminals in the striatum 2 weeks after neurotoxin administration by using TH-immunohistochemistry and neurochemical analysis of DA and its metabolites. LPS alone failed to alter DA levels in the striatum as compared with control non-lesioned animals (Figures 10A,B,E). MPTP treatment largely decreased DA levels (about 40% control levels; Figures 10C,E). Combined LPS/MPTP further decreased DA levels (about 25% control levels; Figures 10D,E), but failed to reach statistical significance, thus suggesting compensatory mechanisms at the dopaminergic terminal.

## Discussion

In this study we demonstrate that peripheral inflammation induced by a single i.p. injection of LPS sensitizes microglia in the nigrostriatal system in response to MPTP, a well-established model of PD in terms of strong up-regulation of galectin-3, a recently identified microglial disease-associated phenotypic marker (Keren-Shaul et al., 2017; Krasemann et al., 2017; Mathys et al., 2017), and classical pro-inflammatory neurotoxic factors. These microglial changes were accompanied by induction of A1 reactive neurotoxic astrocytes in the nigrostriatal system, and, importantly, preceding dopaminergic neurodegenerative events. Notably, peripheral inflammation and MPTP acted synergistically to induce BBB breakdown and enhanced loss of the nigrostriatal dopaminergic system.

Increasing evidence supports a deleterious role of peripheral inflammation in different neurodegenerative diseases (Herrera et al., 2015). In order to demonstrate our hypothesis, microglial activation was first analyzed in terms of density and morphological features in striatum and SN at 12 h, 24 h, and 2 weeks after LPS/MPTP treatment. Our results show that LPS-induced peripheral inflammation increases the number of microglial cells in both SN and striatum after the treatment with MPTP in a time-dependent manner, peaking at 12 h after treatment. Moreover, at this time point most cells showed a typical morphology of activated microglia. However, Iba1-labeled microglia fail to provide clues about the phenotypic nature of one or even different subsets of polarized microglia. Recent transcriptomic studies have characterized the molecular signature of microglia to uncover different microglial activation states associated to aging (Holtman et al., 2015; Galatro et al., 2017) and disease (Keren-Shaul et al., 2017; Krasemann et al., 2017; Mathys et al., 2017). Transcriptional profiles of isolated microglia from different mouse models of aging and different neurodegenerative diseases have been analyzed and found a strikingly similar transcriptional network in all of them (Holtman et al., 2015). All these conditions showed strong up-regulation of Lgals3 (galectin-3) and very interestingly, it was identified as a major instrumental hub gene in driving the previously referred activated microglia phenotype (Holtman et al., 2015). Recent transcriptomic studies at the single cell level have indeed identified a common microglia disease-associated phenotype (Keren-Shaul et al., 2017; Krasemann et al., 2017; Mathys et al., 2017). A remarkable feature in this microglial phenotype is a strong up-regulation of galectin-3 (Keren-Shaul et al., 2017; Krasemann et al., 2017; Mathys et al., 2017). Taken together, galectin-3 emerges as a good marker to label the disease-associated microglia phenotype. Systemic LPS has been shown to induce a classical M1-like pro-inflammatory activation state different to that seen under disease conditions (Keren-Shaul et al., 2017; Krasemann et al., 2017; Mathys et al., 2017). Supporting this, we failed to detect galectin-3 expression in microglial cells in response to systemic LPS injection, even though microglia exhibited typical morphological features of activation. The same was true after MPTP challenge, at least at the post-injection time examined (12 h). Remarkably, combination of systemic LPS and MPTP robustly up-regulated galectin-3 mRNA-expressing microglial cells in the nigrostriatal system, an indication of a switch from homeostatic to disease-associated phenotype preceding the appearance of neurodegenerative events in nigral dopaminergic neurons.

At present, it is evident the potential ability of microglia to show a graded phenotypic polarization or even different polarization states. Consequently, we also analyzed different pro-inflammatory mediators, including TNF-α, which is considered one of the most significant deleterious players in the context of dopaminergic degeneration (Blaylock, 2017). As expected, our results demonstrate that LPS alone is able to induce an increase in the expression levels of several pro-inflammatory cytokines in both the striatum and SN, thus supporting the existence of a microglia pro-inflammatory phenotype in response to systemic LPS injection. From the different experimental conditions tested, it was evident that combined systemic LPS and MPTP treatment led to a more intense pro-inflammatory response in the nigrostriatal system. Especially relevant was the dramatic increase of TNF-α mRNA expression in the ventral mesencephalon (25-fold higher than that seen after either LPS or MPTP treatment). A synergistic effect of peripheral inflammation and MPTP in up-regulating TNF-α was also observed in the striatum, the two key brain areas in dopaminergic neurodegeneration. The inflammatory cytokines TNF-α and IL-1α are major components of the neuroinflammatory response in PD pathogenesis (Sriram et al., 2006; Godoy et al., 2008; McCoy and Tansey, 2008; De Lella Ezcurra et al., 2010; Harms et al., 2012). Interestingly, TNF-α has also been shown to play a critical role in inducing BBB leakage following MPTP administration in terms of extravasation of FITC-labeled albumin (Zhao et al., 2007). BBB integrity is critical in preventing brain entrance of environmental factors, neurotoxic blood-derived products and peripheral cells and associated inflammatory response and neurodegenerative conditions (Sweeney et al., 2018). Moreover, there is evidence supporting a dysfunctional BBB in PD (Kortekaas et al., 2005). Consequently, we analyzed BBB integrity. It is important to state that serum protein leakage in response to MPTP has been analyzed in mice (Brochard et al., 2009) and found a transient BBB disruption (detectable at 6 h but not at 12 h after MPTP treatment). We analyzed BBB disruption in terms of IgG extravasation at 12 h, and hence comparable to that seen by Brochard et al. (2009). In keeping with this study, we found absence of BBB leakage in striatum and SN at 12 h when MPTP was administered alone. LPS-induced systemic inflammation also failed to alter BBB integrity in terms of IgG extravasation. Interestingly, combined LPS/MPTP triggered a quite robust BBB disruption, thus highlighting the critical deleterious effect of systemic inflammation under conditions of brain injury. It could be argued that local effect of LPS in the peritoneal cavity could increase absorption of MPTP and hence underlying some of the differences seen between MPTP and LPS + MPTP groups. This is, however, unlikely illustrated by the lack of a graded response in terms of BBB disruption when comparing MPTP group (no effect at 12 h) with LPS + MPTP group (robust effect at 12 h). Altogether, our study supports the view that systemic LPS and MPTP act synergistically.

Recent evidence has shown that astroglia also seem to play an important deleterious role in neurodegenerative diseases. Hence, two different types of reactive astrocytes, termed A1 and A2 in analogy to the M1/M2 macrophage nomenclature, have been recently described (Zamanian et al., 2012; Liddelow et al., 2017). While A1 astrocytes up-regulate genes previously shown to be harmful, A2 astrocytes are protective through the up-regulation of many neurotrophic factors. Pro-inflammatory microglia are the cell responsible for driving astrocytes from a resting state to the A1 phenotype (Liddelow et al., 2017). Once we have demonstrated that peripheral inflammation sensitizes microglia to acquire both, a disease-associated and a pro-inflammatory phenotype in response to MPTP, we wondered if this effect was accompanied by induction of astrogliosis with the neurotoxic A1 astrocytic phenotype, which express high levels of the C3 component of the complement cascade (Liddelow et al., 2017). Our immunofluorescence study clearly revealed that treatment with both, LPS and MPTP, is a very potent inducer of A1 neurotoxic astrocytes in the nigrostriatal dopaminergic system. This finding highlights the critical importance of peripheral inflammation in driving both, microglia and astroglia, into potentially neurotoxic phenotypes *in vivo*. Consequently, we next wanted to know if this neuroinflammatory scenario could increase dopaminergic neuronal death in the ventral mesencephalon following MPTP injection. Our results showed that systemic LPS exacerbates damage to the dopaminergic system in the ventral mesencephalon in response to MPTP, thus confirming the prominent role of peripheral inflammation in regulating brain neurotoxic glia response to subsequent damage to the nigrostriatal dopaminergic system.

Our study revitalizes the importance of peripheral inflammation in the context of neurodegeneration, and particularly PD (Herrera et al., 2015). We provide evidence that conditions associated to a peripheral inflammatory process may constitute a risk factor for PD. In addition, considering that the BBB is a potential gateway to the environment, we demonstrate that peripheral inflammation ease this interaction. It should be noted that exposure to environmental neurotoxins is assumed to be a significant contributor to nerve cell loss (neurodegeneration) in certain locations in the midbrain of PD patients. Consequently, our findings may be important in the context of PD etiology as environmental exposure is considered an accepted risk factor for PD, from which, pesticide exposure showed the strongest association (Kalia and Lang, 2015).

## Author Contributions

JV and RMD: conceptualization. IG-D, KV, JG-R, AC-J, MR-C, MS, and RMD: investigation. IG-D, KV, JG-R, AC-J, and MS: methodology. IG-D and RMD: formal analysis. JV, RMD, and IG-D: writing – review and editing. JV and RMD: funding acquisition and supervision.

## Conflict of Interest Statement

The authors declare that the research was conducted in the absence of any commercial or financial relationships that could be construed as a potential conflict of interest.

## Footnotes

1 https://recursoshumanos.us.es/index.php?page=seprus

2 http://rsb.info.nih.gov/ij/download.html

## Supplementary Material

FIGURE S1 Early Analysis (24 h) of integrity of nigral dopaminergic neurons and appearance of cleaved caspase-3 in the ventral mesencephalon after LPS/MPTP treatment. Immunofluorescence of TH **(A,D,G,J)**, and cleaved caspase-3 **(B,E,H,K)**. Merge images **(C,F,I,L)**. Note the appearance of cleaved caspase-3 in response to combined LPS/MPTP treatment; also note that dopaminergic neurons failed to co-localize with cleaved caspase-3. Scale bar: 200 μm.

FIGURE S2 Occludin immunostaining in a control animal. Normal pattern of occludin is seen in striatum **(A)** and SN **(C)**. Occludin immunostaining in a LPS/MPTP treated animal. 12 h after the injection of both LPS and MPTP. Images show a clear decrease in the immunoreactivity of occludin in the striatum **(B)** and SN **(D)**, especially in the areas surrounding the vessels.

FIGURE S3 Normal pattern of IgG immunoreactivity in control animals in Cortex **(A)** and Midbrain (adjacent to superior colliculus) **(B)**. Note the absence of IgG immunoreactivity. IgG extravasation is induced 12 h after the combination of LPS and MPTP. Note the atypical presence of IgG immunostaining in cortex **(C)** and midbrain **(D)**. Scale bar: 100 μm.
