# Supplementary figures and images for: Peripheral Inflammation Enhances Microglia Response and Nigral Dopaminergic Cell Death in an in vivo MPTP Model of Parkinson’s Disease
Source: Front Cell Neurosci. 2018 Nov 6;12:398. doi: 10.3389/fncel.2018.00398 (PMC6232526; doi:10.3389/fncel.2018.00398)

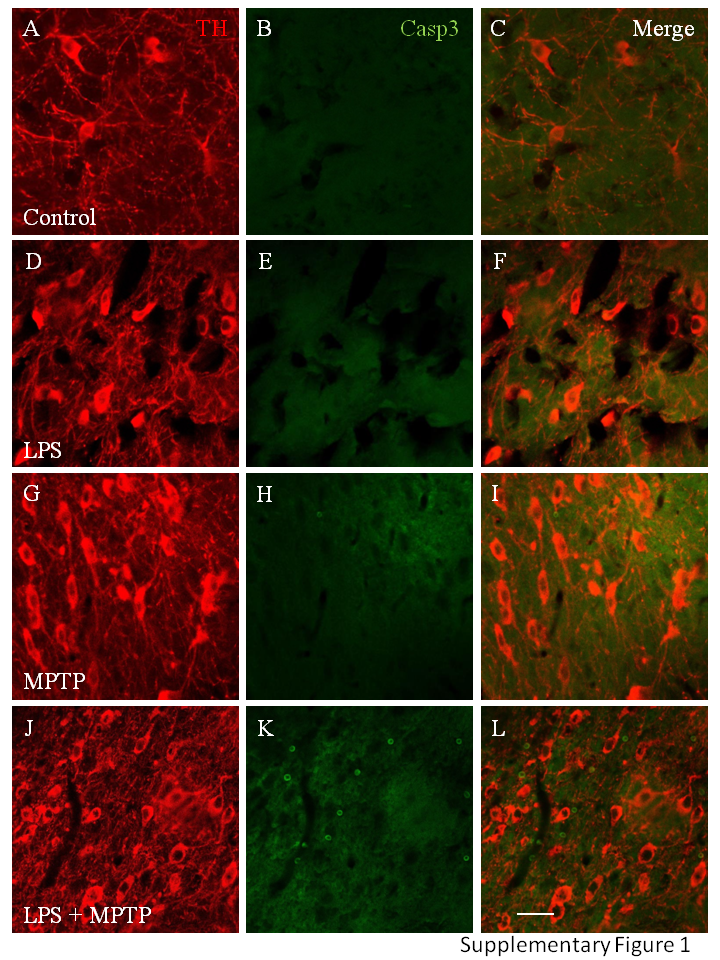

Supplement: FIGURE S1 — Early Analysis (24 h) of integrity of nigral dopaminergic neurons and appearance of cleaved caspase-3 in the ventral mesencephalon after LPS/MPTP treatment. Immunofluorescence of TH (A,D,G,J), and cleaved caspase-3 (B,E,H,K). Merge images (C,F,I,L). Note the appearance of cleaved caspase-3 in response to combined LPS/MPTP treatment; also note that dopaminergic neurons failed to co-localize with cleaved caspase-3. Scale bar: 200 μm. [file Image_1.TIF]

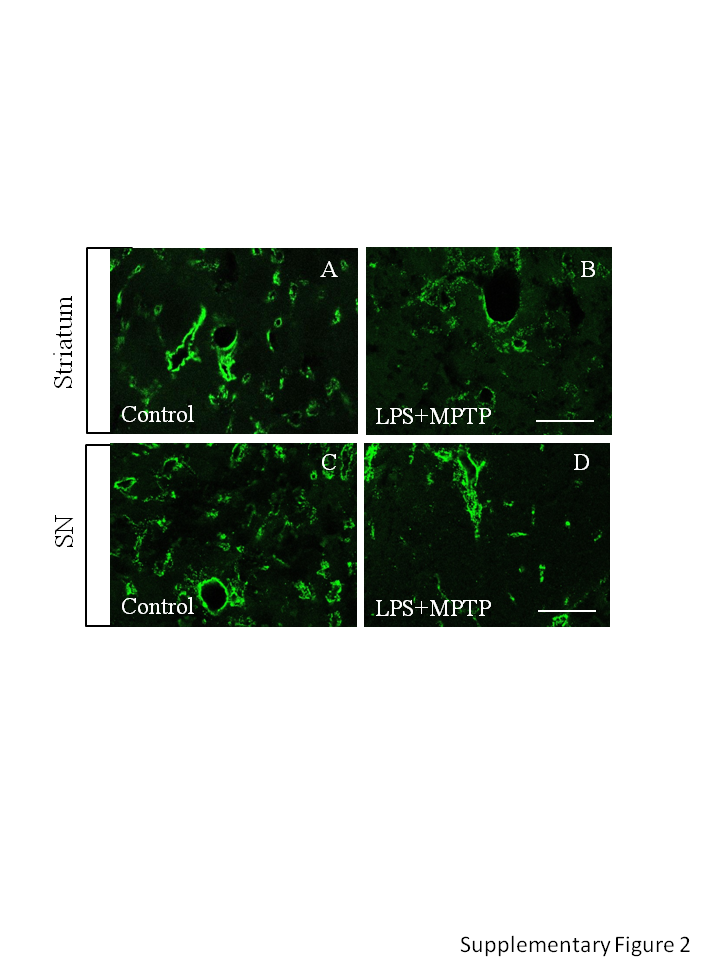

Supplement: FIGURE S2 — Occludin immunostaining in a control animal. Normal pattern of occludin is seen in striatum (A) and SN (C). Occludin immunostaining in a LPS/MPTP treated animal. 12 h after the injection of both LPS and MPTP. Images show a clear decrease in the immunoreactivity of occludin in the striatum (B) and SN (D), especially in the areas surrounding the vessels. [file Image_2.TIF]

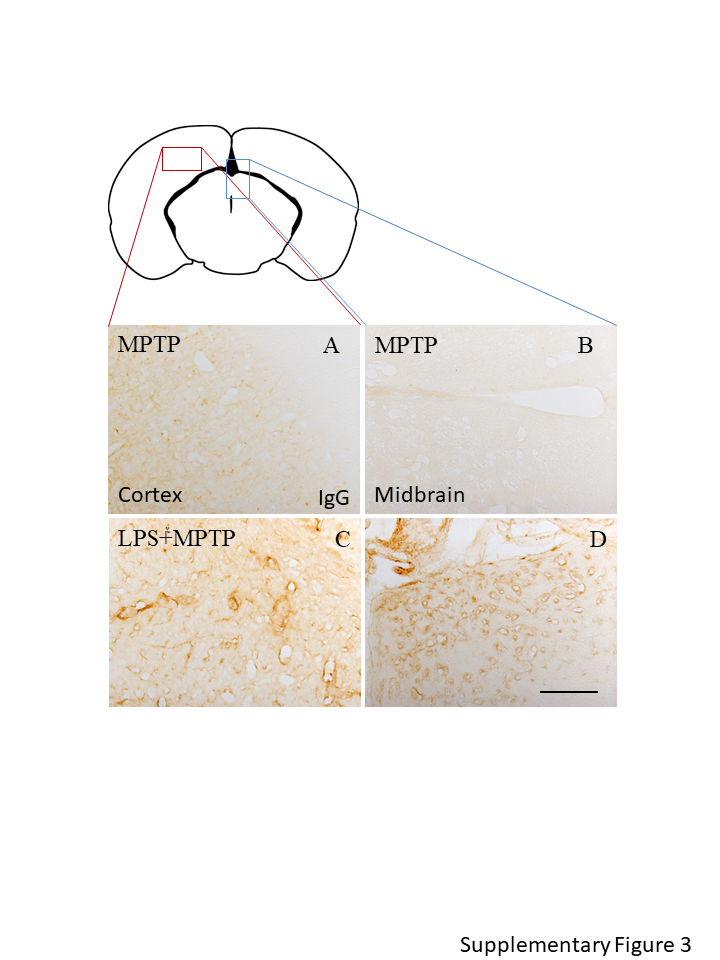

Supplement: FIGURE S3 — Normal pattern of IgG immunoreactivity in control animals in Cortex (A) and Midbrain (adjacent to superior colliculus) (B). Note the absence of IgG immunoreactivity. IgG extravasation is induced 12 h after the combination of LPS and MPTP. Note the atypical presence of IgG immunostaining in cortex (C) and midbrain (D). Scale bar: 100 μm. [file Image_3.TIF]
